# Ranavirosis in Invasive Bullfrogs, Belgium

**DOI:** 10.3201/eid1712.110236

**Published:** 2011-12

**Authors:** Mojdeh Sharifian-Fard, Frank Pasmans, Connie Adriaensen, Sander Devisscher, Tim Adriaens, Gerald Louette, An Martel

**Affiliations:** Ghent University, Merelbeke, Belgium (M. Sharifian-Fard, F. Pasmans, C. Adriaensen, A. Martel);; Research Institute for Nature and Forest, Brussels, Belgium (S. Devisscher, T. Adriaens, G. Louette)

**Keywords:** ranavirus, ranavirosis, *Lithobates catesbeianus*, invasive species, viruses, bullfrogs

**To the Editor:** Massive global declines in amphibians have been attributed to various causes, including infectious diseases such as chytridiomycosis and ranavirosis. Chytridiomycosis and ranaviral disease are international notifiable diseases because they have been listed by the World Organisation for Animal Health in its Animal Health Code.

Ranavirosis is caused by icosahedral cytoplasmic DNA viruses that belong to the family *Iridoviridae*, in particular by 4 species of *Ranavirus*: Frog Virus 3 (FV3), Bohle iridovirus, *Ambystoma tigrinum* virus, and a possible species *Rana catesbeiana* virus Z. In Europe, FV3 has been identified in several outbreaks of ranavirosis, characterized by mass deaths, notably in green frogs (*Pelophylax* sp.) in Denmark, Croatia, and the Netherlands (*1**,**2*); *Rana temporaria* and *Bufo bufo* in the United Kingdom (*3**,**4*); and *Alytes obstetricans* and *Ichthyosaura alpestris* in Spain (*5*). The invasive exotic bullfrog (*Lithobates catesbeianus*) has been introduced in several European countries and has established large breeding populations in France, Italy, Germany, Greece, and Belgium (*6*).

In addition to their direct effect on native amphibians through competition and predation, bullfrogs are thought to be carriers of chytridiomycosis (*7**,**8*) and, possibly, ranaviruses. Although mass deaths of *L. catesbeianus* tadpoles has been reported in aquaculture facilities, *L. catesbeianus* tadpoles are generally considered a subclinical reservoir of ranaviruses in the United States (*9*).

To assess the role of bullfrogs as carriers of ranaviruses in Europe, we collected 400 clinically healthy tadpoles of *L. catesbeianus* from 3 invasive bullfrog populations at Hoogstraten, Belgium (51°47′Ν, 4°75′Ε) during May–June 2010. All larvae were euthanized as part of an invasive species eradication project and stored at –20°C until further use. At necropsy, liver tissues were collected, and DNA was extracted by using the Genomic DNA Mini Kit (BIOLINE, London, UK). PCR to detect ranavirus was performed as described by Mao et al. (*10*).

Three samples showed positive results with this PCR. These samples were sequenced by using primers M4 and M5 described by Mao et al. (*10*) and blasted in GenBank. A 100% homology with the common midwife toad (*A. obstetricans*) ranavirus partial major capsid protein gene (GenBank accession no. FM213466.1) was found (*5*). Despite the low prevalence of *Ranavirus* infection (0.75%) in the bullfrog tadpoles examined, this study shows that invasive bullfrogs, a known reservoir of chytridiomycosis, are also a likely carrier of ranaviral disease in Europe.
